# Stretch reflex excitability in contralateral limbs of stroke survivors is higher than in matched controls

**DOI:** 10.1186/s12984-019-0623-8

**Published:** 2019-12-05

**Authors:** Taimoor Afzal, Matthieu K. Chardon, William Z. Rymer, Nina L. Suresh

**Affiliations:** 1 Department of Physical Medicine and Rehabilitation, Northwestern University, 355 E. Erie Street, Floor 21, Chicago, IL 60611 USA; 2 Single Motor Unit Lab, Shirley Ryan AbilityLab, 355 E. Erie Street, Floor 21, Chicago, IL 60611 USA; 3 Department of Physiology, Northwestern University, Chicago, IL USA

**Keywords:** Stroke, Motoneuron excitability, Reticulospinal tract, Electromyography, Tendon-tapping

## Abstract

**Background:**

Spasticity, characterized by hyperreflexia, is a motor impairment that can arise following a hemispheric stroke. While the neural mechanisms underlying spasticity in chronic stroke survivors are unknown, one probable cause of hyperreflexia is increased motoneuron (MN) excitability. Potential sources of increased spinal MN excitability after a stroke include increased vestibulospinal (VS) and/or reticulospinal (RS) drive. Spasticity, as clinically assessed in stroke survivors, is highly lateralized, thus RS contributions to stroke-induced spasticity are more difficult to reconcile, as RS nuclei routinely project bilaterally to the spinal cord. Yet studies in stroke survivors suggest that there may also be changes in neuromodulation at the spinal level, indicative of RS tract influence. We hypothesize that after hemispheric stroke, alterations in the excitability of the RS nuclei affect both sides of the spinal cord, and thereby contribute to increased MN excitability on both paretic/spastic and contralateral sides of stroke survivors, as compared to neurologically intact subjects.

**Methods:**

We estimated stretch reflex thresholds of the biceps brachii (BB) muscle using a position-feedback controlled linear motor to progressively indent the BB distal tendon in both spastic and contralateral limbs of hemispheric stroke survivors and in age-matched intact subjects.

**Results:**

Our previously reported results show a significant difference between reflex thresholds of spastic and contralateral limbs of stroke survivors recorded from BB-medial (*p* < 0.005) and BB-lateral (*p* < 0.001). For this study, we report that there is also a significant difference between the reflex thresholds in the contralateral limb of stroke subjects and the dominant arm of intact subjects, again measured from both BB-medial (*p* < 0.05) and BB-lateral (*p* < 0.05).

**Conclusion:**

The reduction in stretch reflex thresholds in the contralateral limb of stroke survivors, based here on comparisons with thresholds of intact subjects, suggests an increased MN excitability on contralateral sides of stroke survivors as compared to intact subjects. This in turn supports our contention that RS tract activation, which has bilateral descending influences, is at least partially responsible for increased stretch reflex excitability, post-stroke, as both contralateral and affected sides show increased MN excitability as compared to intact subjects. Still, spasticity, presently diagnosed only on the affected side, with increased MN excitability on the affected side as compared to the contralateral side (our previous study), may be due to a different strongly lateralized pathway, such as the VS tract, which has not been directly tested here. Currently available clinical methods of spasticity assessment, such as the Modified Ashworth Scale, lack the resolution to quantify this phenomenon of a bilateral increase in MN excitability.

## Background

Spasticity is a frequent consequence of hemispheric stroke, in which contralesional muscles demonstrate exaggerated stretch reflex responses. About one-third of stroke survivors develop this physical sign [1]. We now believe that spasticity is a manifestation of increased motoneuron (MN) excitability, and that this increased excitability is due primarily to a sustained depolarization of the membrane potential of MNs innervating spastic muscles. This means that there is a reduction in necessary muscle afferent driven synaptic input required to reach the motoneuron excitation threshold [2]. Thus, a reduction in the stretch reflex threshold is a key finding in examining the sources of increased motoneuron excitability. In addition to reduced membrane potentials, suppression of presynaptic inhibitory mechanisms has also been proposed as a cause of spasticity [3, 4].

To establish an association between MN excitability and spasticity, it is essential to address the role of the descending pathways in modulating the stretch reflex. It is well established that excitatory and inhibitory signals of supraspinal origin are responsible for the regulation of excitability of the stretch reflex pathway [3, 5, 6]. Evidence from invasive animal lesion studies indicates that among the major descending tracts i.e., corticospinal, reticulospinal (RS), vestibulospinal (VS), rubrospinal, and tectospinal, only RS and VS tracts, originating in the brainstem, are substantially involved in the regulation of spinal stretch reflex circuitry [3]. Similar findings have been reported in humans with no neurological disorder. Corticospinal and rubrospinal tracts have limited influence on stretch reflex excitability [3], and the tectospinal tract appears not to exist in humans [7]. Thus, potential sources of increased spinal MN excitability after a stroke include increased VS and/or RS drive, potentially due to disruption of cortico-bulbar inhibitory pathways projecting to these brainstem nuclei. While animal studies have provided evidence of brainstem participation in spasticity, analogous direct approaches in humans to assess brainstem activity have not been possible [8]. The role of the RS tract in the pathophysiology of spasticity in humans has been examined with the acoustic startle reflex (ASR) studies in stroke survivors. ASR is a brainstem-mediated reflex that has been utilized as a non-invasive tool to examine the RS activity in humans [9–11]. In these studies, an exaggerated ASR response has been observed in spastic muscles of chronic stroke survivors as compared to the contralateral limbs of stroke survivors and control subjects [10, 12]. Specifically, RS tract contributions have been thoroughly studied in fast hand extension movements [13], ballistic movements [14] and two-dimensional reaching tasks [15] in stroke subjects.

Hyperexcitability of RS tract provides increased neuromodulatory input to the spinal cord. A well-studied source of neuromodulation to MNs is the raphe pathway descending from the raphe nuclei in the brainstem and that releases the key monoamine, serotonin. Anatomical studies have consistently shown near symmetrical bilateral projections of these RS pathways [16, 17]. Furthermore, hyperexcitability of the RS tract could lead to increased motor overflow i.e., unilateral voluntary activation could elicit activation of the contralateral limb [8], indicating the bilateral influence of the RS tract. Thus, a change in excitability of brainstem RS centers is likely to impact both sides of the body.

The pivotal issue in addressing the effects of the RS tract in humans is the sharp lateralization of clinical spasticity in stroke survivors. Accordingly, the objective of our study is to quantitatively assess whether there is also an increased MN excitability on the contralateral side of stroke survivors, in comparison to matched neurologically intact subjects. If so, this would help us evaluate the contributing role of the RS tract towards spasticity, and help us quantify its putative magnitude. Our goal is to utilize precision reflex measurement techniques to characterize and compare changes in (deep tendon) reflex behavior in both the spastic and contralateral muscles of stroke survivors and neurologically intact subjects. We propose that reflex threshold measures could be used as a surrogate estimate of MN excitability and thereby enable the characterization of the impact of the bilaterally distributed systems such as the RS tract and other brainstem monoaminergic pathways.

## Methods

### Participants

We recruited nine hemiparetic spastic stroke survivors (6 males, 3 females) and ten age-matched intact subjects (5 males, 5 females), serving as controls. The age range of stroke survivors was 49–79 years and the age range of intact subjects was 45–71 years. The inclusion criteria for the stroke survivors were a single hemispheric stroke occurring at least 6 months before participation in the study. Further criteria required the subjects to show a spasticity level > =1 on the Modified Ashworth Scale (MAS). For the stroke survivors, testing was conducted on two separate days, with the spastic limb tested before the contralateral limb. Testing on the control subjects was conducted in a single session on their dominant arm only. All participants gave informed consent through the study protocol approved by the Institutional Review Board under the Office for the Protection of Subjects at Northwestern University. The data recorded from stroke subjects has been previously presented in [18].

### Experimental setup

A detailed description of the experimental setup can be found in [18]. Here, we briefly review the experimental setup. The key experimental device consists of a position-controlled linear actuator with a resolution of 5 μm (Linmot S.A., Spreitenbach, Switzerland). At the end of the linear slider, a 1 degree of freedom load cell is attached. To facilitate the positioning of the device and to achieve precise repeatability of tendon tapping, the device was mounted on a custom frame with three linear and three rotational degrees of freedom. One linear degree of freedom was controlled using a manual micrometer with a capability of providing accuracy as high as a tenth of a millimeter (Velmex, Bloomfield, NY, USA). Position correction of the linmot was performed by a PID controller, internal to the device. The controller was tuned to achieve a rise time, settling time, and overshoot of 3 ms, 10 ms, and 8% respectively.

**Fig. 1:**
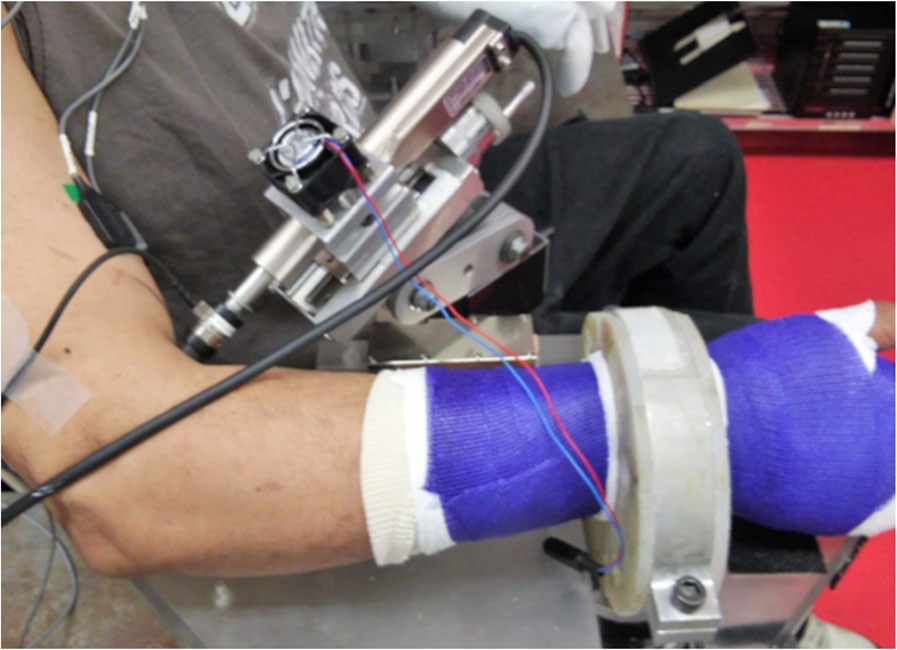
Experimental Setup. The subject is seated with the arm flexed. The Linmot is positioned perpendicular to the distal biceps tendon with the tip in contact with the skin surface. The arm is casted and clamped at the wrist and resting on a flat surface at the elbow to maintain a steady position. EMG sensing electrodes are placed on the biceps muscle (Electrode on the lateral head is visible).

Subjects were seated on a Biodex chair and the trunk was secured by Velcro straps across their torso from shoulder to hip. The wrist and forearm on the testing side were casted and clamped at the wrist to a custom magnetic base mounted on a steel table. The tapper’s long axis was positioned at 90^o^ to the biceps brachii’s (BB) distal tendon. The starting position was manually zeroed through visual inspection to begin indentation on the surface of the skin. To ensure repeatability, the contact of the tapper was continuously monitored on a computer screen via the load cell attached to the end. Electromyogram (EMG) signals were recorded using active bipolar surface electrodes (Delsys, Boston, MA, USA) placed on the medial and lateral heads of the BB and the triceps brachii. Before electrode placement, the skin was cleaned with alcohol pads. The experimental setup is shown in Fig. 1.

**Fig. 2:**
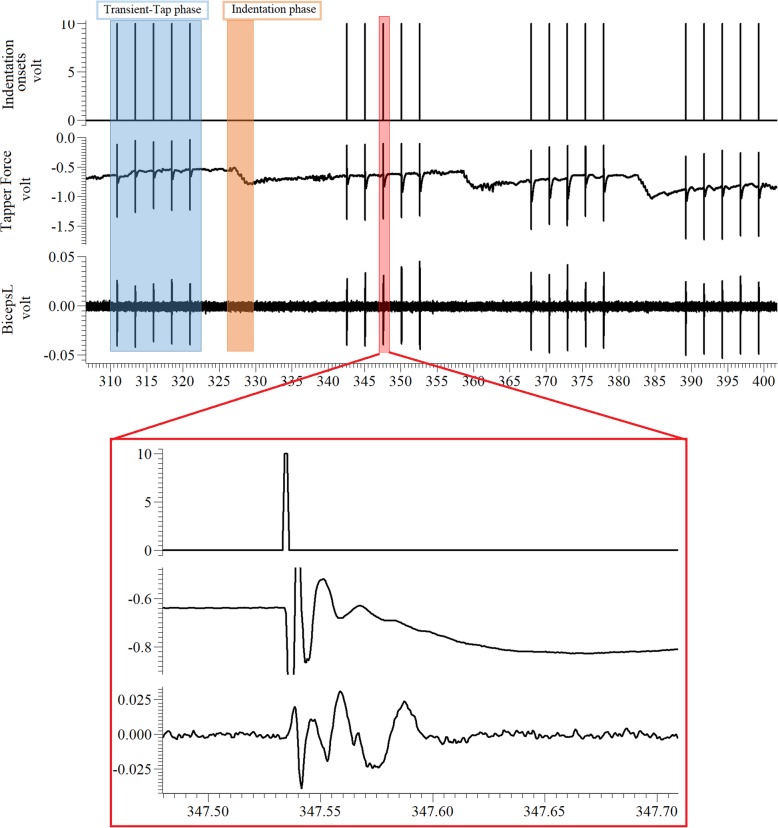
Raw data (from Spike 2) showing the indentation sequence, force response as measured from the load cell at the tip of the slider, and EMG measured from the BB-lateral muscle. Each spike corresponds to one tap. The force gradually increases (negative value indicates increase) with each tendon indentation of 1 mm. The expanded view shows a sample tap with 1 mm depth and the corresponding force and EMG response.

The tapping protocol consisted of two phases, an indentation phase (orange window in Fig. 2, top trace) and a transient-tap phase (blue window in Fig. 2, top trace). The indentation phase would change the resting depth of the tip of the device with a slow “ramp and hold” indentation, using increments of 1 mm starting at the skin (i.e. 0, 1, 2, 3 … mm). The micrometer was used to perform the slow indentation. Once the indentation phase was over, the transient-tap phase began from each new resting depth location. It consisted of five taps of 1 mm magnitude, separated by 2.5 s. This cycle was repeated until the maximum indentation depth of 25 mm was reached or up to a level of discomfort that was just tolerated by our subjects (see Table 1). The subjects were specifically instructed to relax during the protocol and EMG of the subjects was monitored after each indentation phase to ensure that it returned to baseline. No adverse events occurred during any of the sessions.

**Table 1:** Threshold and maximum indentation of stroke subjects in the spastic (S) and contralateral (C) sides and on the dominant side of control subjects as calculated from the EMG activity recorded from the BB muscle (medial and lateral). The values in bold are the measured thresholds. For subjects with no identifiable threshold, the maximum indentation was assigned as the threshold value for further analysis

Stroke							Control			
Sub. #	Threshold (mm)				Maximum Indentation (mm)		Sub. #	Threshold (mm)		Maximum Indentation (mm)
	BB-Medial		BB-Lateral					BB-Medial	BB-Lateral	
	S	C	S	C	S	C				
1	**5**	**13**	**4**	**13**	20	20	1	25	25	25
2	**4**	**9**	**6**	**9**	23	24	2	**14**	18	18
3	**4**	**16**	**10**	**16**	24	25	3	23	23	23
4	**6**	**20**	**11**	**20**	14	25	4	19	19	19
5	**8**	**11**	**7**	**14**	21	19	5	25	25	25
6	**10**	**15**	**12**	**22**	21	25	6	24	24	24
7	**13**	25	**10**	25	25	25	7	25	25	25
8	**7**	25	**9**	**21**	23	25	8	21	21	21
9	**10**	**12**	**10**	**12**	18	25	9	20	**16**	20
							10	**18**	**17**	23
Mean ± SD	7.4 ± 3.1	16.2 ± 5.9	8.8 ± 2.6	16.9 ± 5.3	21.0 ± 3.4	23.7 ± 2.4		21.4 ± 3.7	21.3 ± 3.6	22.3 ± 2.6

The compression force between the tissue and the actuator tip was recorded using the load cell attached to the tip of the linear actuator. This force measurement represents the “pushback” force from the muscle and subcutaneous tissue. Force data were low pass filtered (Butterworth with cut-off 600 Hz) and sampled at 1 kHz (Power 1401, CED, Cambridge, U.K.). EMG signals were recorded using a Delsys Bagnoli system (Boston, MA, USA). The raw EMG was filtered (20–450 Hz) then amplified by a gain of 100. The surface EMG signals were sampled at 2 kHz (Power 1401, CED, Cambridge, U.K.). The software package Spike2 (ver. 6) was used to collect and synchronize all the signals and control the recording sessions. The indentation sequence, force, and EMG traces are shown in Fig. 2.

### Data analysis

#### Biceps Brachii EMG activity

To quantify the level of EMG activity in the BB muscles (lateral and medial heads), we computed the rectified-integrated EMG (RIEMG) of the response. The activity was extracted using a window of 50 ms duration, beginning 20 ms after the tendon tap. It has been shown that the reflex component begins 15–20 ms after the tap [18]. The pre EMG activity was estimated using the RIEMG extracted from a 50 ms window preceding the tendon tap.

#### Estimation of reflex threshold

The reflex threshold was designated as the first indentation depth at which at least 3/5 taps resulted in post RIEMG responses greater than mean + 3 standard deviations (SD) of the pre-tap RIEMG activity for at least 3 consecutive indentation positions.

#### Statistical analysis

We used paired sample t-test to compare the reflex threshold between the spastic and contralateral limbs of the stroke subjects. We used unpaired sample t-test to compare the reflex threshold from the control subjects with reflex threshold from the spastic and contralateral limbs of stroke subjects. The tolerance level was set to 5%.

## Results

The objective of this study was to quantify the differences in the reflex threshold between spastic and contralateral BB muscle in stroke survivors, and between the contralateral arm in stroke survivors and the dominant arm of age-matched intact subjects, serving as controls. In all stroke survivors, we were able to record a definitive indentation depth as a representation of the reflex threshold on the spastic limb. We also observed an increase in the EMG response magnitude as the indentation increased beyond the indentation threshold, thereby meeting the reflex threshold criteria.

### Indentation depth similarity: stroke vs. control

As the reflex response is highly dependent on the indentation depth, we ensured that the tendon was indented to a similar depth, i.e., 25 mm for each subject. However, there was some variation in the maximum indented depths across subjects, for a couple of reasons. In some cases, the underlying tissue mass prevented full-depth indentation, and in some cases, the subjects were unable to tolerate indentation beyond a certain depth. Despite this variability, the average maximum indentation depths between different sides were comparable. The average maximum indentation was 21.0 ± 3.4 mm on the spastic limb and 23.7 ± 2.4 mm on the contralateral limb. For control subjects, the average maximum indentation was 22.3 ± 2.6 mm. We therefore found no significant differences in the maximum indentation depths available between the spastic and contralateral limb. In addition, there was no significant difference in the maximum depths achieved between the spastic and control limbs, and between contralateral and control sides. The recorded reflex threshold, as measured from the BB-medial and BB-lateral muscles, and the maximum indentation depths for each subject are shown in Table 1.

### Effect of indentation position on the reflex response

**Fig. 3:**
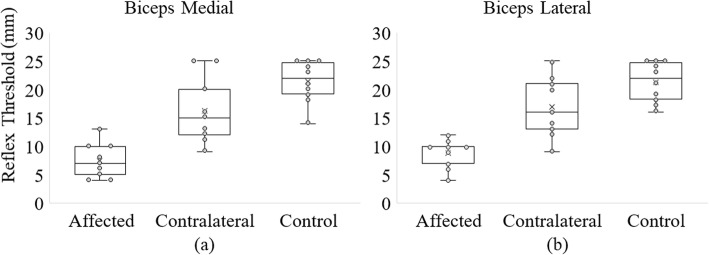
Reflex threshold measured from the BB muscle **a** Medial and **b** Lateral. The reflex threshold is lowest on the stroke subjects’ spastic side, intermediate for the stroke subjects’ contralateral side and highest for control subjects. The dots represent individual subjects’ reflex thresholds. The ‘x’ represents the mean reflex threshold.

The reflex thresholds measured from the medial and lateral head of the BB muscle are represented as box plots in Fig. 3 and the values are also given in Table 1. The reflex threshold was measurable in 9/9 stroke subjects from the EMG in the BB-medial and BB-lateral muscle in the spastic side, in 7/9 subjects on the contralateral side from BB-medial muscle and in 8/9 subjects on the contralateral side from BB-lateral muscle.

In control subjects, 2/10 subjects showed a measurable reflex threshold from the BB-medial and the BB-lateral muscle. Interestingly, for only a single control subject (subject 10) the reflex threshold was detected from both the BB-medial and lateral muscle. For control subject 2, the reduced reflex threshold was recorded only on the BB-medial muscle. Similarly, for control subject 9, the reflex threshold criterion was satisfied only on the BB-lateral muscle. For subjects with no identified threshold, the maximum indentation depth was assigned as the threshold value for further analysis. We assign a reflex threshold, even when one is not detected, for comparative purposes. In these cases, we were unable to indent further due to discomfort of the tested subject. Our assumption is that with further indentation, a reflex response would have been detected. Thus, the reflex threshold we report is lower than the ‘real’ threshold. For comparative purposes, this will only reduce our potential for statistical significance.

The mean reflex threshold of all stroke survivors as measured from the BB-medial muscle was 7.4 ± 3.1 mm on the spastic limb and 16.2 ± 5.9 mm on the contralateral limb. We found a significant difference between the reflex thresholds on the spastic and contralateral sides measured from the BB-medial EMG (*p* < 0.005). For control subjects, the mean reflex threshold was 21.4 ± 3.7 mm on their dominant limb, as recorded from the BB-medial muscle. We also found a significant difference in the mean reflex thresholds between the spastic and control sides (*p* < 0.001), and between the contralateral and control sides (*p* < 0.05).

The mean reflex threshold for stroke survivors from the BB-lateral muscle was 8.8 ± 2.6 mm on the spastic limb and 16.9 ± 5.3 mm on the contralateral limb. We found a significant difference between the reflex thresholds of the spastic and contralateral sides measured from the BB-lateral muscle (*p* < 0.001). For control subjects, the mean reflex threshold was 21.3 ± 3.6 mm on their tested dominant limb measured from the BB-lateral muscle. We also found a significant difference between spastic and control sides (p < 0.001) and between the contralateral and control sides (*p* < 0.05) as measured from the BB-lateral muscle.

**Fig. 4:**
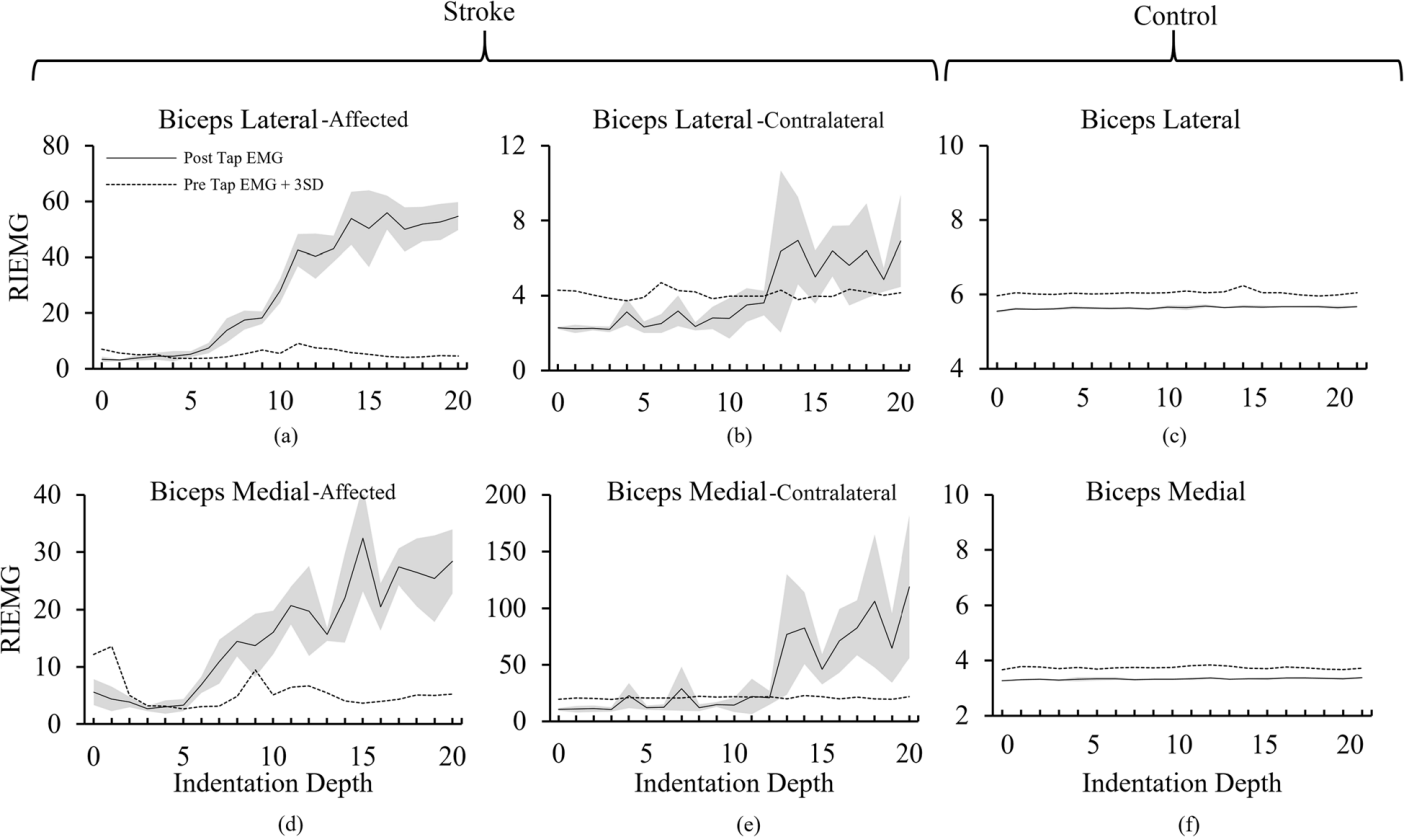
EMG plots from two exemplar subjects. **a**-**b** EMG responses in the spastic and contralateral BB-lateral muscle of one stroke subject. **c** EMG response from the BB-lateral of the dominant arm of one control subject. **d**-**e** EMG responses in the spastic and contralateral BB-medial muscle of the same stroke subject. **f** EMG response from the BB-medial of the dominant arm of the same control subject. The reflex threshold for the spastic side was 4 mm as measured from the BB-lateral (**a**) and 5 mm as measured from the BB-medial muscle (**d**). The reflex threshold for the contralateral side was 13 mm (**b** and **e**). No reflex response was detected for the control subject despite tapping the tendon at an indentation of 24 mm (**c** and **f**). Note the different scales.

The average RIEMG activity of the five taps at each indentation depth for an exemplar stroke subject and an exemplar control subject is shown in Fig. 4. The EMG activity crosses the mean + 3SD of the pre-EMG activity in both the spastic and contralateral sides of the stroke subject Fig. 4 (a), (b), (d) and (e). The reflex threshold for the exemplar stroke subject is 5 mm measured from the BB-medial muscle and 4 mm from the BB-lateral muscle. The reflex threshold on the contralateral side is 13 mm, much greater than that found on the spastic side. For the exemplar control subject, the EMG response magnitude never reaches a value greater than the mean + 3SD of the baseline (Fig. 4(c) & (f)) thus the reflex threshold remain undetected. A summary of statistical results is given in Table 2.

**Table 2:** Statistical results

Muscle	Indentation threshold comparison	*P* values
BB-Medial	Stroke-spastic vs Stroke-contralateral	< 0.01
	Stroke-spastic vs Control	< 0.001
	Stroke-contralateral vs Control	< 0.05
BB-Lateral	Stroke-spastic vs Stroke-contralateral	< 0.001
	Stroke-spastic vs Control	< 0.001
	Stroke contralateral vs Control	< 0.05

#### Correlation of reflex threshold: spastic vs. contralateral

**Fig. 5:**
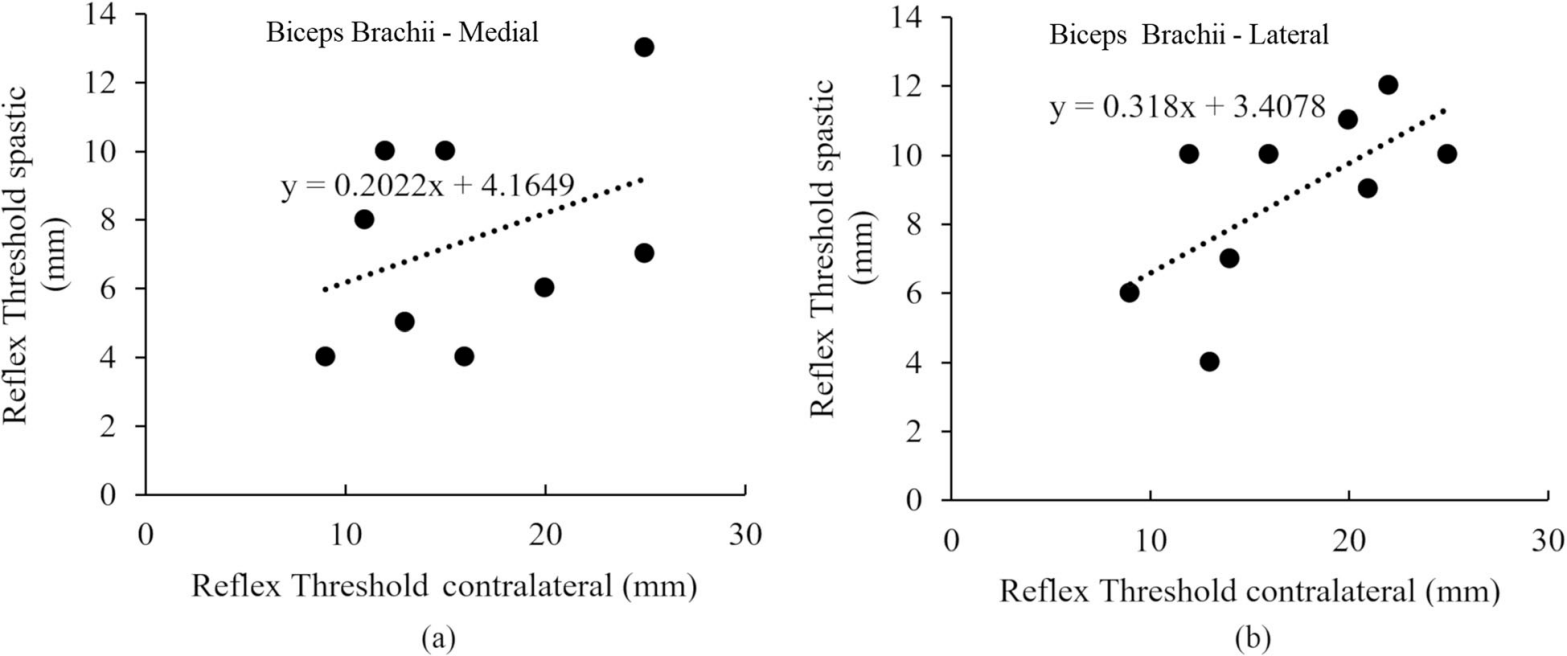
Correlation of reflex threshold between the spastic limb and contralateral limb as measured from BB **a** Medial and **b** Lateral

The correlation of the reflex threshold for the spastic and contralateral limbs is shown in Fig. 5. We observed a moderate positive correlation (*r* = 0.48) of the reflex threshold measured from the BB-medial and strong positive correlation (*r* = 0.66) in reflex threshold measured from the BB-lateral between the spastic and contralateral sides.

## Discussion

In this study, we utilized a position-feedback controlled linear slider for tendon tapping to accurately estimate the stretch reflex thresholds in the dominant arm of control subjects. We then compared our results to reflex thresholds obtained in the spastic and contralateral BB muscle of chronic stroke survivors from a prior study. As reported previously, a significantly lower reflex threshold was observed on the spastic limb of stroke subjects compared to that on the contralateral limb [18]. The novel result of this study is that the reflex threshold on the contralateral limb was significantly lower than that found in the BB muscle in the dominant arm of age-matched intact subjects.

These statistically significant differences in reflex threshold between the contralateral limb of stroke and the dominant limb of intact subjects support our hypothesis that after stroke there is a bilateral impact on MN excitability in both limbs of our sample of stroke survivors, potentially due to stroke-related damage of bilaterally distributed spinal tracts (such as the RS tract). Besides, correlation analysis of the reflex thresholds between the spastic and contralateral sides showed a clear positive correlation (Fig. 5). This observation suggests that the RS influence is proportional bilaterally, i.e., the reduced threshold on the contralateral side is a function of the excitability on the spastic side.

In previous work, particularly in animal studies, it appears that there is a bilateral near-symmetrical distribution of the RS tract within the spinal cord. It follows that any influence of the RS tract on stretch reflexes should be discernable on both sides after a hemispheric stroke. Axons containing serotonin, originating in neurons within the caudal portion of raphe nucleus [19], are known to project densely throughout both sides of the cord. These neuromodulatory inputs alter the intrinsic properties of MNs to produce an increase in their excitability by enhancing voltage-gated conductances for sodium (MN soma) and calcium (MN dendrites). Cortical damage in stroke appears to alter the control of these modulating pathways in the brainstem [18].

### Acoustic startle reflex (ASR)

The ASR has been used to estimate the involvement of the RS tract in stroke survivors, and the results suggest a contributing role of the RS tract to spasticity. These studies also report a prevalence of exaggerated startle reflex on the spastic side in stroke survivors compared to the contralateral side during rest [10, 12]. Jankelowitz and Colebatch also reported a lower latency in the BB of the spastic limb compared to the contralateral limb at rest [10]. Previously, findings from animal studies have consistently supported the essential role of the caudal pontine reticular nucleus in the startle reflex with a lesion to this area inhibiting the startle reflex [20]. The ASR circuitry involves the cochlear nucleus, MNs of the brainstem, caudal pontine reticular nuclei, and the spinal cord activated through the medial RS tract [21]. Exaggerated ASR responses have been observed in the spastic muscle of chronic stroke survivors suggesting that there is RS tract hyperexcitability compared to the contralateral limb of stroke survivors and control subjects [10, 12]. However in these studies systematic comparisons between the contralateral limbs of hemispheric stroke survivors and control subjects were not performed, thus the bilateral effect of RS tract hyperexcitability has not been documented.

### Lateralization of spasticity

Our data supports the contention that RS tract activation, which has bilateral descending influences, is at least partially responsible for increased stretch reflex excitability, post-stroke, as both contralateral and affected sides show increased MN excitability as compared to intact subjects. Still, spasticity, diagnosed routinely only on the affected side, with strongly increased MN excitability on the affected side as compared to the contralateral side (our previous study), may be due to a more strongly lateralized pathway, such as the VS pathway. Below, we highlight possible mechanisms that should further enhance our understanding, including research studies that show unequal distributions of the RS tract.

One plausible explanation of the sharp lateralization of spasticity can be the involvement of the VS tract. Miller et al. found significant differences in the relative amounts of the vestibular drive impacting the MN pools in the spastic and contralateral sides [22]. These differences have been attributed to the disruption of inhibitory corticobulbar projections to the contralesional vestibular nuclei. However, the role of RS tract in spasticity cannot be fully ignored as the vestibular and reticular complexes do have extensive interconnections [23]. It has been suggested that acoustic stimuli could also activate ASR through the RS pathways [8] but results from animal studies show that the lesion to the vestibular nuclei fails to eliminate startle [20]. Further investigation is necessary to observe the isolated effect of acoustic stimuli to the vestibular nuclei on MN pools in the absence of RS tract influence.

It could be argued that despite being extended bilaterally and substantially affecting the MN excitability on both sides of the cord, the differences in the reflex threshold or the sharp lateralization of spasticity may be due to unequal distribution of the neurons projecting in the RS tract. This could result in increased MN excitability on both sides of the cord as compared to intact subjects, with greater influence on the affected side. Evidence to support this claim originates from studies investigating the anatomical distribution of RS projections [24, 25]. It is not yet possible to establish the precise anatomical distribution of RS projection in humans using tracing studies, but evidence from animal studies indicates that bilateral distribution of the neurons on the RS tract is not exactly equal to both sides.

For example, Sakai et al. have used bilateral retrograde tracing in non-human primates to show a regional variation in the distribution of RS neurons in the ponto-medullary reticular formation [24]. Further, they utilized unilateral retrograde tracing to determine the ipsilateral and contralateral contributions of the pontine-medullary reticular formation. They found a dominant proportion (60:40) of the RS cells arising from the ipsilateral gigantocellular reticular nucleus, similar distributions of neurons arising from the Caudal pontine-reticular nucleus (10 times less in number than those found in gigantocellular reticular nuclei), and higher contralateral contributions arising from the Oralis pontine-reticular nucleus. Based on this, it would be a reasonable assumption that despite being bilateral, the distribution of neurons in the RS pathway may have a weak ipsilateral dominance, which could explain the difference in reflex threshold between the contralateral and spastic limbs in this study. However, it is important to mention that the criteria to identify the reticular nuclei are generally made on gross features such as cell size and density, thus the boundaries of these nuclei are not necessarily distinct [16]. Therefore, we consider it is inconclusive to associate the sharp lateralization of spasticity to the weak ipsilateral dominance of neurons distribution.

Another factor that may contribute to increased MN hyperexcitability on the spastic side is activation of persistent inward currents, due to the possible increase in monoaminergic drive to the spinal cord. The neural circuitry and mechanisms by which this could occur on the spastic side only are yet to be determined. While the role of persistent inward currents in spasticity after spinal level lesions has been well established, there is limited evidence that supports the role of persistent inward currents and monoamines in spasticity after a cerebral lesion [26].

Clinically practiced methods to objectively measure spasticity, such as MAS evaluation, require minimal time to perform but lack the resolution to identify subtle changes in MN excitability [27]. While the linear-motor setup is time-consuming, the method used in this paper and previously in [18, 28] is more reliable in quantifying changes in MN excitability. Further, this method provides a consistent accuracy at the millimeter scale that could be utilized when measuring outcomes over the time course of treatment.

## Conclusion

We conclude that increased MN excitability is manifested bilaterally in stroke survivors with spasticity, as characterized by a decrease in the reflex threshold on both the spastic and contralateral sides as compared to neurologically intact subjects. However, there still exists a large reflex threshold differential between the spastic and contralateral sides of tested stroke survivors. Based on our findings, a laterally projecting tract, particularly the VS tract, could have a more dominant role in the manifestation of spasticity. The tendon indentation method is more precise than other clinically practiced methods to objectively measure spasticity, such as MAS evaluation, as it provides a greater resolution to quantify substantial changes in MN excitability.

## Data Availability

The datasets used and/or analyzed during the current study are available from the corresponding author on reasonable request.

## Abbreviations

ASR: Acoustic Startle Reflex
BB: Biceps Brachii
EMG: Electromyography
MAS: Modified Ashworth scale
MN: Motoneuron
RIEMG: Rectified-integrated EMG
RS: Reticulospinal
SD: Standard deviation
VS: Vestibulospinal
